# The complete mitochondrial genome of Slender Giant Moray *Strophidon sathete* (Hamilton, 1822)

**DOI:** 10.1080/23802359.2021.1915720

**Published:** 2021-07-06

**Authors:** Wei Tan, Yongbo Wang, Hongji Ke, Hongtao Liu

**Affiliations:** a Ministry of Education, Key Laboratory of Utilization and Conservation for Tropical Marine Bioresources (Hainan Tropical Ocean University), Sanya, China; b Hainan Provincial Key Laboratory of Tropical Maricultural Technologies, Hainan Academy of Ocean and Fisheries Sciences, Haikou, China

**Keywords:** *Strophidon sathete*, mitochondrial genome, phylogenetic analysis

## Abstract

The whole mitochondrial genome of the Slender Giant Moray *Strophidon sathete* (Hamilton, 1822) from the Hainan island was characterized using next-generation sequencing for the first time. The circular mitogenome of *S. sathete* is 16,568 bp, with 13 protein-coding genes (PCGs), 22 tRNA genes, two rRNA genes, and a D-loop region. The base composition is little biased (A, G, T, and C was 30.95%, 16.73%, 27.09%, and 25.23%, respectively) with A + T contents of 58.04%. Among 13 PCGs, 12 PCGs use a normal ATG as the start codon except COX1 use GTG; four of them end with TAA or TAG, others terminate with an unusual stop codon. The phylogenetic tree showed that *S. sathete* was first clustered with *Rhinomuraena quaesita* and *Gymnothorax minor*, which further clarify the phylogenetic and evolution position of the genus *Strophidon* in the family Muraenidae.

*Strophidon sathete*, commonly known as Slender Giant Moray or Gangetic Moray, belonging to the family Muraenidae, is the largest member of the family of moray eels. It is broadly distributed across the Indo-West Pacific from South Africa, India, Malaysia, Indonesia, Taiwan eastwards to Australia (Chen et al. 1994; Loh et al. 2015). It inhabits muddy areas in the lower reaches of rivers, estuaries, and coastal marine waters (Böhlke 1997) and feeds mainly on a variety of small fishes and crustaceans. It is marketed fresh and consumed locally. The nomenclature and taxonomic history of species within *Strophidon* are contentious and its members are easily misidentified (Huang et al. 2020). Till now, mitochondrial genome records of *Strophidon* species are inadequate, the mitogenome of *S. sathete* we provided was the first reported. In the present study, the complete mitochondrial genome analysis of the spiny spooner will help to investigate the relationships among the genera and species of the family Muraenidae.

The samples of the slender giant moray were collected from Dongying, Haikou, China (N 20°3′5.0148″, E 110°26′46.014″), and stored in the Hainan Marine Science and Technology Museum (Hongtao Liu, xmulht@gmail.com) under the voucher number F20191230SS in Qionghai research base of Hainan Academy of Ocean and Fisheries Sciences. The library with an average length of 350 bp was constructed using the NexteraXT DNA Library Preparation Kit, and sequencing was performed on the Illumina Novaseq platform (Total Genomics Solution Limited, SZHT) for the 150 bp average length of the generated reads. The whole mitochondrial genome assembled 1.97 G raw reads using the GetOrganelle v1.6.2e (Jin et al. 2020), and annotated using the MITOS (http://mitos.bioinf.uni-leipzig.de/index.py). A phylogenetic analysis was carried out based on the 13 PCGs encoded by 13 mitogenomes available in GenBank using IQ-TREE v1.6.12 (Nguyen et al. 2015) by maximum-likelihood (ML) method with 1000 bootstrap replicates, the Best-fit model is mtVer + F+R4 chosen according to Bayesian information criterion (BIC).

The complete mitogenome of the slender giant moray submitted to the GenBank database (accession no.: MW035594) is 16,568 bp in length. The base composition is 30.95% A, 16.73% G, 27.09% T, and 25.23% C. The 58.04% of (A + T) shows a little higher preference than G + C. It consists of 13 protein-coding genes (PCGs), 22 tRNA, two rRNA, and a D-loop region. ND6 gene, eight tRNA, and the D-loop region are located on the light strand, the others are encoded by the heavy strand.

The 22 tRNA genes in the mitogenome of the slender giant moray vary from 67 bp to 76 bp. Two tRNA are present more than once: tRNA-Leu and tRNA-Ser both have two type copies, respectively. The 12S rRNA is 949 bp, located between tRNA-Val and tRNA-Phe; the 16S rRNA is 1660 bp, located between tRNA-Val and tRNA-Leu. There are six overlapping regions of 1–9 bp in length. The longest overlapping region is located between ATP8 and ATP6. The mitochondrial genome has 16 intergenic sequences varying from 1 to 915 bp in length (Supply Table S1). The largest intergenic sequence is the D-loop region located between tRNA-Pro and tRNA-Phe. Twelve PCGs use a normal ATG as the initiation codon except COX1 start with GTG. Four genes (ND1, ND4L, ND6, CYTB) end with TAA or TAG, other genes terminate with unusual stop codon: ATP8, ATP6, and COX3 use TA; ND2 and COX2 use T; COX1 uses AGA; ND3 uses GAA; ND4 uses TGT; ND5 uses ACC.

The phylogenetic tree (Figure 1) showed that *S. sathete* was first clustered with *Rhinomuraena quaesita* and *Gymnothorax minor*, which further elucidate the phylogenetic and taxonomic position of the genus *Strophidon* in the family Muraenidae. The results are largely similar to previous studies based on mitochondrial COI fragments, 12S and 16S rRNA genes (Tang and Fielitz 2013; Huang et al. 2020). Taken together, the newly sequenced mitochondrial genome of *S. sathete* characterized here should contribute to a better understanding of phylogenetic relationships of species in the family Muraenidae, and molecular identification, population genetic and evolutionary biological studies of the slender giant moray.

**Figure 1.**
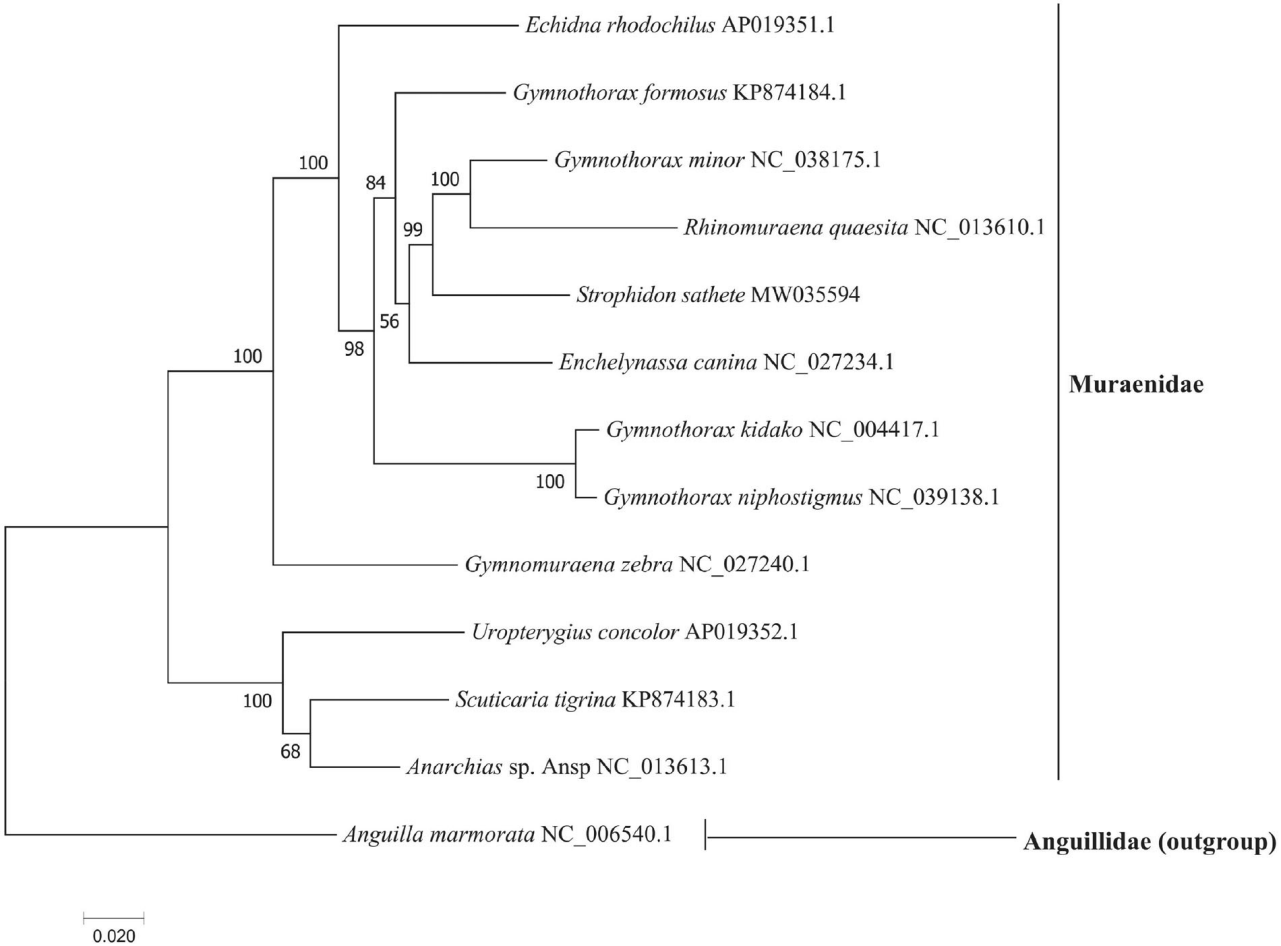
Phylogenetic tree of the complete mitogenome of 13 fish species in Anguilliformes.

The GenBank accession number for each species is indicated after the scientific name. *Anguilla marmorata* was used as outgroup.

## Data Availability

The genome sequence data that support the findings of this study are openly available in GenBank of NCBI at https://www.ncbi.nlm.nih.gov/ under the accession no. MW035594. The associated BioProject, SRA, and Bio-Sample numbers are PRJNA703745, SRR13758977, and SAMN18022891, respectively.

## References

[CIT0001] Böhlke EB. 1997. Notes on the identity of elongate unpatterned Indo-Pacific morays, with description of a new species (Muraenidae, Subfamily Muraninae). Proc Acad Nat Sci Philadelphia. 147:89–109.

[CIT0002] Chen H-M, Shao K-T, Chen C-T. 1994. A review of the muraenid eels (family Muraenidae) from Taiwan with descriptions of twelve new records. Zool Stud. 33:44–64.

[CIT0003] Huang WC, Mohapatra A, Thu PT, Chen HM, Liao TY. 2020. A review of the genus Strophidon (Anguilliformes: Muraenidae), with description of a new species. J Fish Biol. 97:1462–1480. 32844437 10.1111/jfb.14514

[CIT0004] Jin J-J, Yu W-B, Yang J-B, Song Y, Depamphilis CW, Yi T-S, Li D-Z. 2020. GetOrganelle: a fast and versatile toolkit for accurate de novo assembly of organelle genomes. Genome Biol. 21(1):31. 32912315 10.1186/s13059-020-02154-5 PMC7488116

[CIT0005] Loh K-H, Hussein MAS, Chong V-C, Sasekumar A. 2015. Notes on the moray eels (Anguilliformes: Muraenidae) of Malaysia with two new records. Sains Malay. 44:41–47.

[CIT0006] Nguyen L-T, Schmidt HA, Von Haeseler A, Minh BQ. 2015. IQ-TREE: a fast and effective stochastic algorithm for estimating maximum-likelihood phylogenies. Mol Biol Evol. 32:268–274. 25371430 10.1093/molbev/msu300 PMC4271533

[CIT0007] Tang KL, Fielitz C. 2013. Phylogeny of moray eels (Anguilliformes: Muraenidae), with a revised classification of true eels (Teleostei: Elopomorpha: Anguilliformes). Mitochondrial DNA. 24:55–66. 22967094 10.3109/19401736.2012.710226

